# On the Likelihood of Surrogates Conforming to the Substituted Judgment Standard When Making End-of-Life Decisions for Their Partner

**DOI:** 10.1177/0272989X19862800

**Published:** 2019-07-29

**Authors:** Eleonore Batteux, Eamonn Ferguson, Richard J. Tunney

**Affiliations:** University of Nottingham, Nottingham, UK; University of Nottingham, Nottingham, UK; Aston University, Birmingham, UK

**Keywords:** aging, end of life, substituted judgment standard, surrogate decision making

## Abstract

A considerable proportion of end-of-life decisions are made by the patient’s next-of-kin, who can be asked to follow the substituted judgment standard and decide based on the patient’s wishes. The question of whether these surrogate decision makers are actually able to do so has become an important issue. In this study, we examined how the likelihood of surrogates conforming to the substituted judgment standard varies with individual differences in mortality acceptance and confidence in their decision making. We recruited 153 participants in romantic relationships between 18 and 80 years old from the general population. We asked them to make hypothetical end-of-life decisions for themselves and on behalf of their partner, as well as predict what their partner would do, and complete a series of questionnaires. Participants predicted that their partner would make similar decisions to their own but were more likely to accept a life-saving treatment that could result in reduced quality of life on their partner’s behalf than for themselves. Decisions made by older adults were more likely to conform to the substituted judgment standard, which is encouraging given that they are more likely to be confronted with these decisions in real life, although this was not due to differences in mortality acceptance. Older adults were also more likely to have had previous discussions with their partner and thereby know that person’s wishes and feel confident that they made the right decision, but these factors did not affect their likelihood of conforming to the substituted judgment standard. This shows that encouraging discussions about end of life among families would ease the decision process, but more work is needed to ensure that surrogates can adhere to the substituted judgment standard.

More than 70% of deaths in intensive care units (ICU) are the result of decisions to withhold or withdraw life-sustaining treatment, but only about 5% of patients are able to make these decisions for themselves.^1^ In these circumstances, it is common for a next-of-kin to act as a surrogate decision maker. They are often instructed to follow the substituted judgment standard, whereby they must make a decision based on their knowledge of the patient’s preferences. This varies according to each country’s legislation. It is the case in the United States that surrogates are required to follow the substituted judgment standard. In the United Kingdom, they are instructed to consider both the patient’s wishes and his or her best interests. However, doubts have been cast on the suitability of the substituted judgment standard, given that it assumes that surrogates are able to decide according to the patient’s preferences.

The question of whether surrogates can accurately predict their next-of-kin’s wishes has been extensively posed. A systematic review of the literature has found that surrogate accuracy is around 68%,^2^ meaning that a significant proportion of surrogate decision makers did not meet the substituted judgment standard. A second question that has arisen is whether surrogates do make their decisions according to their predictions of the surrogates’ preferences or whether they choose differently. In this article, we investigate whether a range of factors affects surrogates’ propensity to make a decision that conforms with the substituted judgment standard in end-of-life scenarios.

Tunney and Ziegler’s model^3^ of surrogate decision making assumes that the decision maker engages in perspective taking, which varies according to particular features of the decision (see Figure 1). Surrogates try to adopt the perspective that matches the required benchmark when making end-of-life decisions, given that they are highly significant decisions for which they could be held accountable. If surrogates are instructed to follow the substituted judgment standard, they should engage in simulated perspective taking (predicting what the recipient *would* do). Simulation historically refers to the psychological ability to put oneself in other people’s shoes to predict their behavior.^4^ This requires acknowledging the differences between the surrogate and the recipient to simulate what they would have done. The substituted judgment standard expects that surrogates take a simulated perspective when making their decision. A simulated decision would be a decision that conforms to the surrogate’s predictions of what the recipient would have done. However, they might also follow a benevolent perspective (what the recipient *should* do) to preserve the recipient’s best interests or engage in an egocentric perspective (what the surrogate wants) to preserve their own interest. Finally, surrogates might rely on a projected perspective (what the surrogate would do in the recipient’s situation) if in doubt about the recipient’s preferences. This is different from the simulated perspective in that it does not take any differences between the surrogate and the recipient into account. The model therefore assumes that surrogates can be prevented from strictly adhering to the substituted judgment standard, even if they intend to make a simulated decision. What can previous research tell us about the way surrogates make end-of-life decisions?

**Figure 1:**
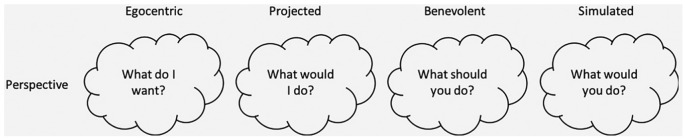
Tunney and Ziegler’s model^3^ of surrogate decision making in which the surrogate considers various perspectives to make a choice.

Studies that have investigated whether decision making on behalf of other people differs from decisions made for ourselves have found that we are more likely to avoid taking high risks for others. This has been shown both when medical professionals make decisions for patients^5,6^ and when people from the general population decide for a stranger or family member.^7–9^ Irrespective of the illness or treatment in question, surrogates are more likely to favor the option that is most likely to preserve the patient’s life. When deciding for themselves, people are more inclined to accept or refuse a treatment that could increase their chances of dying to avoid an illness^10^ or complications from a treatment.^6^

These findings have been interpreted as surrogates being more cautious when deciding for someone else, rather than surrogates believing that the recipients would also be more cautious for themselves. Surrogate decisions have in fact been shown to differ from surrogate predictions—people predict others to take similar risks as they would, but surrogates take fewer risks for others than for themselves.^11,12^ On the other hand, in a within-subjects design, we found that surrogate predictions were significant predictors of surrogate choices, independently of the decision maker’s own choices.^13^ This suggests that surrogates do not simply disregard the recipient’s preferences but are influenced by other factors.

Qualitative reports of surrogates’ experiences after the fact confirm that they intend to follow the substituted judgment standard.^14–16^ They draw on their knowledge of the recipient’s wishes, which reassures them that they are making the right decision, but struggle to ignore other factors. For example, surrogates feel a responsibility to preserve the patient’s life and the family’s well-being. They also find it difficult to disregard their own wishes for their loved one (i.e., that they do not want to lose them). This confirms suspicions that the substituted judgment standard is difficult to meet and is usually not adhered to in reality.

A recent mixed-methods study^17^ revealed a number of factors that affect surrogates’ propensity to make a simulated decision (i.e., decide based on their knowledge of the recipient’s wishes). Older adult partners were asked to make a series of end-of-life decisions for each other before being interviewed about their decision process. Participants were more likely to take a life-sustaining treatment for their partner than their partner did for themselves, thereby resulting in surrogate inaccuracy. However, surrogates reported that they drew on their knowledge of the recipient’s wishes to inform their decisions, which gave them the confidence that they were making the right decision. It seemed to be the case that those who had previous discussions with their partner were more confident, which, in turn, made them more likely to take a simulated perspective. They also seemed more comfortable with mortality and had had experiences of life-threatening illnesses, either themselves or through a close relative. They therefore appeared more prepared to make a decision that would end their partner’s life if they believed those were his or her wishes. In the present study, we drew from this to experimentally investigate how these factors affect surrogates’ propensity to make a simulated decision in end-of-life scenarios and conform to the expectations of the substituted judgment standard.

We recruited participants from the general population and asked them to make hypothetical end-of-life decisions for themselves and their partner. They were also asked for their surrogate predictions (i.e., to indicate what they expect their partner would decide for himself or herself). This allowed us to compare their surrogate decisions to their surrogate predictions to evaluate the extent to which they made a simulated decision. The more similarities there were between the two, the more participants were considered to have made a simulated decision. They then had to indicate their confidence that they made the right decision, their knowledge of their partner’s wishes, and whether previous discussions on the matter had taken place. We measured their fear of their own and their partner’s death to assess their acceptance of mortality. Finally, participants reported their previous experiences relating to illness and death. We recruited a large range of ages given that these measures are likely to vary with age. We could then assess whether surrogates’ propensity to make a simulated decision for their partner varied with age and length of relationship. This study was preregistered with Open Science Framework (https://osf.io/bsjf8/). Our preregistered hypotheses were as follows:

*Hypothesis 1.* We expected that participants would predict that their partner would make similar end-of-life decisions to their own but would be more willing to make a decision that would lead to their own life ending rather than their partner’s.
*Hypothesis 2.* We expected that older adults would be more likely to have experiences of illness and death, thereby making them more accepting of mortality for themselves (2a) and for their partner (2b). We predicted this to increase their likelihood to refuse treatment, both for themselves (2c) and for their partner (2d).
*Hypothesis 3.* We expected that older adults would be more likely to have experiences of illness and death, thereby making them more accepting of their partner’s mortality. We, therefore, predicted that older adults were more likely to have discussions with their partner, which in turn increases surrogates’ knowledge of their partner’s wishes and confidence that they were making the right decision (3a). We expected this to increase their likelihood of making a simulated decision (3b) and lead to smaller self-partner differences (3c). (We erroneously indicated that this would lead to larger self-other differences in our preregistration form; we expect a higher propensity of a simulated decision to be linked to smaller self-other differences.)
*Hypothesis 4.* We expected longer relationships with a partner to increase previous discussions, knowledge of wishes, and confidence in making the right decision. We predicted that this in turn would increase their likelihood of making a simulated decision.

## Methods

### Design

The study was a within-subjects design where participants made decisions for themselves and their partner, as well as predicted their partner’s wishes. The order in which these were completed was randomized.

### Participants

We recruited participants online via Prolific (https://prolific.ac) from the United Kingdom. Given that we could not derive an estimated effect size for our study based on previous research, we hypothesized that we would find a medium effect size. We conducted a power analysis using G*Power 3.1 to determine the necessary sample size to detect a medium effect size using a multiple linear regression with 7 predictors (to test hypothesis 3). A sample size of 153 is required to detect a medium effect size (*f*^2^ = 0.15) with high power (>.95) and an acceptable α level (<.05). This sample size allows for enough power to test the remainder of our hypotheses: detection of a medium effect (*d* = 0.5) of recipient (hypothesis 1), with high power (>.95) and an acceptable α level (<.05), and mediated effects (hypotheses 2–4), assuming that the α and β paths have medium effect sizes.^18^ We therefore recruited 153 participants who were in a romantic relationship. To obtain a range of ages, we recruited older adults (60–80) separately from younger adults (18–59). Ethical approval was obtained from the University of Nottingham’s ethics committee.

### Decision-Making Task

Participants completed 2 scenarios adapted from the willingness to accept life-sustaining treatment (WALT)^19^ instrument. Each scenario depicted a life-threatening situation in which participants are taken to the hospital for weeks to months. They are offered a high-burden treatment course to recover by a doctor. The probability of the treatment working varied from 90% to 10% in decrements of 10. In each case, participants had to indicate whether they would want the treatment or not. They were told that they would not survive without treatment. Each scenario varied in terms of the outcome of the treatment: either the treatment works and their current health is restored, or the treatment does not work and they end up bedbound (functional impairment scenario) or end up unaware (cognitive impairment scenario). The order in which they completed each scenario was randomized. They completed the task 3 times in a random order: once making decisions for themselves (*self*), once on behalf of their partner (*partner*), and once where they had to predict what they thought their partner would choose (*prediction*). The exact wording of the scenarios can be found in Supplementary File 1.

### Questionnaires

Participants completed a series of questionnaires after the WALT instrument (see Supplementary File 1). They were first asked questions relating to the scenarios they had completed: whether they had previously discussed end-of-life scenarios with their partner (*discussions*), whether they felt like they knew their partners’ wishes (*knowledge*), and how confident they were that they made the right decision for themselves and then for their partner (*confidence*) (on a scale from 1–5). The order in which they were presented with these questions was randomized. As a measure of *fear of their own death* and *fear of their partner’s death*, participants completed a revised version of the Collett-Lester Fear of Death scale version 3.0.^20^ Scale reliability of *fear of their own death* (α = 0.85) and *fear of their partner’s death* (α = 0.81) was good. Finally, they completed a shortened version of the revised Life Stressor Checklist, which included questions specific to *experiences* of illness and death.^21^

### Analysis Procedures

We computed indifference points for each scenario and condition (i.e., the point at which participants were indifferent between accepting or rejecting the treatment). We considered the indifference point to be the average of the 2 probabilities on each side of the crossover point from accepting to refusing the treatment. We then took the average of the indifference point for both scenarios as a measure of willingness to accept treatment for each recipient. We excluded participants who made inconsistent choices (e.g., selecting a treatment with a 40% chance of recovery but not a 100% chance) as we could not compute an indifference point for them. We considered inconsistent choices to be problematic as we assumed that they indicated that the participant did not understand or pay attention to the task (particularly if they selected only 1 option, but it was not a 100% chance of recovery). There is a possibility that inconsistent choices show that the participants were conflicted, but their responses to the task would be difficult to interpret, so we did not analyze their choices further. We chose to compute the indifference point rather than the proportion of times participants selected the treatment option to avoid including participants who may not have understood or paid attention to the task. We subtracted *partner* from *self* to have a measure of self-other differences: positive values meant that participants accepted more treatment for their partner than for themselves. We subtracted *prediction* from *partner* and removed the sign to have a measure of simulation. We then reverse scored it so that higher values meant that surrogate decisions deviated less from surrogate predictions and that surrogates were more likely to have made a simulated decision. For every participant, we summed their scores for each item of the fear of death scales and the life experience scale. We analyzed our data as stated in our preregistration as well as some exploratory analyses, which were all conducted in SPSS (SPSS, Inc., an IBM Company, Chicago, IL). For our correlation analyses, we used Pearson’s *r* for continuous variables and Spearman’s ρ for ordinal variables. All mediation analyses were performed using the PROCESS macro for SPSS.^22^ Effects were calculated for each 5000 bootstrapped samples.

## Results

We recruited 167 participants overall as 6 were excluded for not being in a relationship and 8 were excluded for making choices from which we could not compute an indifference point. All 8 participants we excluded selected a treatment with a lower chance of recovery than 100% but did not select the treatment with a 100% chance of recovery. We assumed that they did not understand or pay attention to the task. We then ended up with 153 participants, as required by our power analysis. Participant characteristics can be found in Table 1.

**Table 1:** Participant Characteristics

Characteristic	Participants
Sex, female, %	54
Age, mean (SD), y	45.63 (21.28)
Young adults (aged 18–34 years), %	41
Middle-aged adults (aged 35–59 years), %	12
Older adults (aged 60–80 years), %	47
Length of relationship, mean (SD), y	20.28 (18.37)
Young adults (aged 18–34 years), mean (SD), y	3.35 (3.99)
Middle-aged adults (aged 35–59 years), mean (SD), y	19.10 (10.22)
Older adults (aged 60–80 years), mean (SD), y	35.17 (14.37)

### Preregistered Analyses

#### Hypothesis 1

We analyzed participants’ treatment choices to investigate hypothesis 1. We entered participants’ indifference points into a repeated-measures analysis of variance (ANOVA) with recipient (self, predict, partner) as a 3-level factor. The main effect of recipient was significant (*F*_2, 304_ = 11.226, *MS_e_* = 163.872, *P* < 0.001,*$\eta_{p}^{2}$* = 0.069) and followed a linear trend (*F*_1, 152_ = 17.943, *MS_e_* = 192.753, *P* < 0.001, *$\eta_{p}^{2}$* = 0.106). Pairwise comparisons showed that participants were more willing to accept treatment for their partner than for themselves (mean difference = 6.72, *P* < 0.001). There was no difference between their own choices and their surrogate predictions (mean difference = 1.89, *P* = 0.211), but participants accepted more treatment for their partner than they predicted their partner would (mean difference = 4.83, *P* < 0.001). Hypothesis 1 was supported by our findings.

#### Hypothesis 2

Age was positively correlated with experiences (*r_s_* = .228, *P* = 0.005). However, experiences were not significantly correlated with fear of their own death (*r_s_* = −.132, *P* = 0.103) or their partner’s death (*r_s_* = −.085, *P* = 0.297). The indirect effect between age and self with experiences and fear of own death as mediators was not significant, nor was the one between age and partner with experiences and fear of partner’s death as mediators (see Supplementary File 2 for the full analysis). Hypothesis 2 was overall not supported, apart from the fact that experiences varied with age.

#### Hypothesis 3a

**Figure 2:**
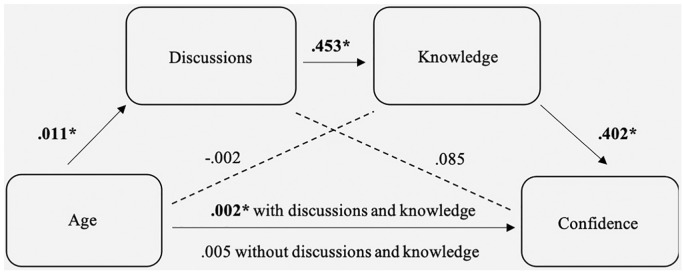
Mediation model showing the relationship between participants’ age and confidence, mediated by previous discussions and knowledge of their partner’s wishes. If significant (*P* < 0.05), unstandardized regression coefficients are denoted with an asterisk. The mediation model was significant.

Age was positively correlated with discussions (*r_s_* = .206, *P* = 0.032). Discussions were positively correlated with knowledge (*r_s_* = .491, *P* < 0.001), and knowledge was positively correlated with confidence (*r_s_* = .547, *P* < 0.001). The mediation analysis examined the link between age and confidence with discussions and knowledge as mediators. The total effect of age on confidence was not significant (*B* = 0.005 [–0.002, 0.012], *SE* = 0.003, *P* = 0.168). The direct effect of age on discussions was significant (*B* = 0.011 [0.003, 0.018], *SE* = 0.038, *P* = 0.006) and accounted for 4.94% of the variance in discussions. The direct effect of age on knowledge was not significant (*B* = −0.002 [–0.008, 0.005], *SE* = 0.003, *P* = 0.599), but discussions on knowledge were (*B* = 0.453 [0.315, 0.592], *SE* = 0.070, *P* < 0.001); age and discussions accounted for 22.2% of the variance in knowledge (*F*_2, 150_ = 21.427, *P* < 0.001). The direct effects of age (*B* = 0.003 [–.004, 0.009], *SE* = 0.003, *P* = 0.400) and discussions (*B* = 0.085 [–0.061, 0.230], *SE* = 0.074, *P* = 0.251) on confidence were not significant, but knowledge was significantly linked to confidence (*B* = 0.402 [0.252, 0.552], *SE* = 0.076, *P* < 0.001); age, discussions, and knowledge accounted for 24.1% of the variance in confidence (*F*_3, 149_ = 15.787, *P* < 0.001). The indirect effect of age on confidence was not significant through discussions (effect = 0.009 [–0.001, 0.003]) or knowledge (effect = −0.001 [–0.003, 0.003]), but it was significant through discussions and knowledge (effect = 0.002 [0.001, 0.004]). See Figure 2 for a representation of the model. Hypothesis 3a, which expected older adults to be more confident they made the right decision due to previous discussions and increased knowledge of their partner’s wishes, was supported.

#### Hypothesis 3b

The likelihood of making a simulated decision was positively correlated with age (*r* = .171, *P* = 0.035): increased age led to a higher likelihood of making a simulated decision. However, simulation was not significantly correlated with discussions (*r_s_* = −.010, *P* = 0.904), knowledge (*r_s_* = .111, *P* = 0.171), or confidence (*r_s_* = .098, *P* = 0.227). The indirect effect between age and simulation with discussions, knowledge, and confidence as mediators was not significant (see Supplementary File 2). Hypotheses 3b was only supported insofar as simulation was linked to age.

#### Hypothesis 3c

**Figure 3:**
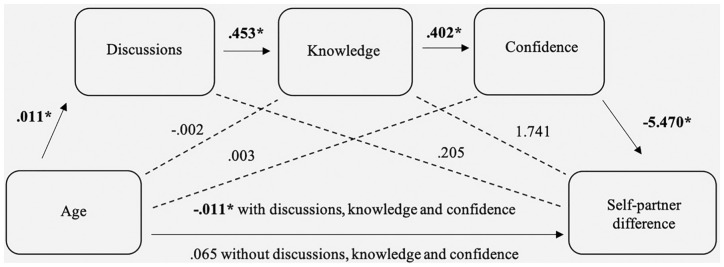
Mediation model showing the relationship between participants’ age and self-partner difference, mediated by previous discussions, knowledge of their partner’s wishes, and their confidence that they made the right surrogate decision. If significant (*P* < 0.05), unstandardized regression coefficients are denoted with an asterisk. The mediation model was significant.

The self-partner difference was not correlated with age (*r_s_* = .046, *P* = 0.576) or discussions (*r_s_* = −.024, *P* = 0.769), but the correlation with knowledge fell short of significance (*r_s_* = −.157, *P* = 0.052). Confidence was negatively correlated with the self-partner difference (*r_s_* = −.213, *P* = 0.008): increased confidence meant participants were less likely to accept more treatment for their partner than for themselves. The mediation analysis examined the link between age and the self-partner difference with discussions, knowledge, and confidence as mediators. The total effect of age on self-partner differences was not significant (*B* = 0.065 [–0.083, 0.213], *SE* = 0.075, *P* = 0.385). The direct effect of age on discussions was significant (*B* = 0.011 [0.003, 0.018], *SE* = 0.038, *P* = 0.006) and accounted for 4.94% of the variance in discussions. The direct effect of age on knowledge was not significant (*B* = −0.002 [–0.008, 0.005], *SE* = 0.003, *P* = 0.599), but discussions on knowledge were (*B* = 0.453 [0.315, 0.592], *SE* = 0.070, *P* < 0.001); age and discussions accounted for 22.2% of the variance in knowledge (*F*_2, 150_ = 21.427, *P* < 0.001). The direct effects of age (*B* = 0.003 [–0.004, 0.009], *SE* = 0.003, *P* = 0.400) and discussions (*B* = 0.085 [–0.061, 0.230], *SE* = 0.074, *P* = 0.251) on confidence were not significant, but knowledge was significantly linked to confidence (*B* = 0.402 [0.252, 0.552], *SE* = 0.076, *P* < 0.001); age, discussions, and knowledge accounted for 24.1% of the variance in confidence (*F*_3, 149_ = 15.787, *P* < 0.001). The direct effect of confidence on self-partner differences was significant (*B* = −5.470 [–9.399, –1.541], *SE* = 1.988, *P* = 0.007), but the direct effects of age (*B* = 0.084 [–0.066, 0.234], *SE* = 0.076, *P* = 0.271), discussions (*B* = 0.205 [–3.342, 3.752], *SE* = 1.795, *P* = 0.909), and knowledge (*B* = 1.741 [–2.229, 5.170], *SE* = 2.009, *P* = 0.388) were not; age, discussions, knowledge, and confidence accounted for 5.43% of the variance in self-other differences (*F*_4, 148_ = 2.126, *P* = 0.080). The indirect effect of age on self-partner differences through discussions, knowledge, and confidence was significant (effect = −0.011 [–0.028, –0.001]), but none of the other indirect effects were. See Figure 3 for a representation of the model. Overall, hypothesis 3c was supported.

#### Hypothesis 4

Length of relationship was positively correlated with discussions (*r_s_* = .218, *P* = 0.007), which were positively correlated with knowledge (*r_s_* = .491, *P* < 0.001), which in turn was positively correlated with confidence (*r_s_* = .547, *P* < 0.001). Simulation was positively correlated with length of relationship (*r* = .193, *P* = 0.017): longer relationships led to a higher likelihood of making a simulated decision. The indirect effect between length of relationship and simulation with discussions, knowledge, and confidence as mediators was not significant (see Supplementary File 2).

#### Regression analysis

We conducted a hierarchical regression analysis with age (step 1), experience and fear of partner’s death (step 2), and discussions, knowledge, and confidence (step 3) as predictors of the likelihood of making a simulated decision. We did not enter length of relationship as a predictor to avoid collinearity problems as it was highly correlated with age (*r* = .865, *P* < 0.001). Step 1 was significant (*F*_1, 151_ = 4.543, *P* = 0.035, *R*^2^ = 0.029), with an increase in age leading to an increase in the likelihood of making a simulated decision (*B* = 0.097, *SE* = 0.046, *P* = 0.035). Step 2 (*F*_3, 149_ = 2.132, *P* = 0.099, *R*^2^ = 0.041) and step 3 (*F*_6, 146_ = 2.903, *P* = 0.057, *R*^2^ = 0.079) fell short of significance. Age was the only variable that consistently predicted simulation. Full results can be found in Table 2.

**Table 2:** Regression Model for Likelihood of Making a Simulated Decision

		*B*	*SE*	*P*
1	Constant	15.710	2.301	<0.001
	Age	0.097	0.046	**0.035**
2	Constant	25.283	7.460	0.001
	Age	0.102	0.048	**0.035**
	Experiences	0.354	0.703	0.615
	Fear of death	0.207	0.161	0.199
3	Constant	38.010	9.313	<0.001
	Age	0.103	0.048	**0.034**
	Experiences	0.429	0.733	0.559
	Fear of death	0.265	0.165	0.111
	Discussions	−1.185	1.152	0.305
	Knowledge	1.661	1.273	0.194
	Confidence	1.684	1.227	0.172

Note: The bold p-values represent statistically significant results at p<.05.

### Exploratory Analyses

#### Treatment choices

To further examine the relationship between surrogate decisions and predictions, we conducted Pearson’s correlations. Surrogate choices and predictions were positively correlated (*r* = 0.860, *P* < 0.001). Partial correlations between surrogate choices and predictions, controlling for participants’ own choices, were significant (*r* = 0.639, *P* < 0.001). We then performed regression analyses to assess whether surrogate predictions were predictive of surrogate choices, independently of participants’ own choices. We found that the model was significant (*F*_2, 150_ = 253.352, *P* < 0.001) and accounted for 77.2% of the variance in surrogate choices. Surrogate predictions significantly predicted surrogate choices (*B* = 0.700, *SE* = 0.069, *P* < 0.001), but so did participants’ own choices (*B* = 0.313, *SE* = 0.069, *P* < 0.001).

#### Discussions

We assessed whether experiences of illness and death had a relationship with surrogates’ propensity to discuss end-of-life scenarios, controlling for age. We found a positive relationship between the two (*r_s_* = .358, *P* < 0.001). We conducted a hierarchical regression analysis with age (step 1) and experience (step 2) as predictors of discussions. Step 1 was significant (*F*_1, 151_ = 7.798, *P* = 0.006, *R*^2^ = 0.049), with any increase in age leading to an increase in discussions (*B* = 0.011, *SE* = 0.004, *P* = 0.006). Step 2 (*F*_2, 150_ = 11.239, *P* < 0.001, *R*^2^ = 0.142) was also significant, with an increase in experience leading to an increase in discussions (*B* = 0.224, *SE* = 0.056, *P* < 0.001). Age was no longer a significant predictor (*B* = 0.007, *SE* = 0.004, *P* = 0.071).

#### Confidence

We conducted a paired-samples *t* test to compare participants’ confidence that they made the right decision for themselves to their confidence that they made the right decision for their partner. We found that participants were significantly more confident for themselves (mean = 4.19, SD = 0.82) than for their partner (mean = 3.86, SD = 0.91) (*t*_1, 152_ = 5.300, *P* < 0.001).

#### Fear of death

We split each scale into the 2 subscales of the original Collet-Lester fear of death scale^20^: the prospect of death itself and the process of dying (see Supplementary File 2). We entered participants’ scores for each subscale into a 2 (person) × 2 (subscale) repeated-measures ANOVA. We found a main effect of person: participants were more fearful of their partner’s death than their own (*F*_1, 152_ = 110.417, *MS_e_* = 12.634, *P* < 0.001, *$\eta_{p}^{2}$* = 0.421). We found a main effect of subscale: participants were more fearful of the process of dying than the prospect of death (*F*_1, 152_ = 23.085, *MS_e_* = 8.376, *P* < 0.001, *$\eta_{p}^{2}$* = 0.132). We also found an interaction between person and subscale (*F*_1, 152_ = 120.889, *MS_e_* = 6.261, *P* < 0.001, *$\eta_{p}^{2}$* = 0.113). Pairwise comparisons showed that participants were more fearful of the process of dying than the prospect of death for themselves (mean difference = −2.013, *P* < 0.001) but not for their partner (mean difference = −0.235, *P* = 0.308). Moreover, we found a negative relationship between age and fear of the prospect of death, both for participants’ own death (*r_s_* = −.299, *P* < 0.001) and their partner’s death (*r_s_* = −.208, *P* = 0.010).

## Discussion

This study sheds new light on the surrogate decision process, including surrogates’ propensity to decide according to their predictions of the recipient’s preferences. We show that previous discussions between partners increase their confidence that they are making the right decision.^a^ This suggests that encouraging people to have discussions earlier about end-of-life preferences would ease the decision process. We also found that surrogates who had been in a relationship for longer were more likely to conform to the substituted judgment standard. They were more likely to have had discussions about end of life, but we did not find that these increased surrogates’ likelihood of deciding according to their predictions of the recipient’s preferences. Although discussions can relieve the burden experienced by surrogate decision makers, they might not successfully reduce surrogate inaccuracy.

As expected, age had an effect on experiences and individual differences relating to mortality: older adults were more frequently exposed to experiences of illness and death and were more likely to fear the prospect of their own and their partner’s death. Age also had an effect on the process of making a surrogate decision: older adults were more likely to have discussions about end of life with their partners, which can be attributed to their previous experiences of illness and death. Notably, having prior discussions increased surrogates’ knowledge of their partner’s wishes and their confidence that, from their perspective, they were making the right decision. These findings shed light on the process of making a surrogate decision, which seems to be eased by having these prior discussions and feeling like one knows the recipient’s wishes. Crucially, this shows that participants hold a conception of the right decision as being related to making a decision in line with the substituted judgment standard. This lends support to its validity as an ethical framework.

The finding that participants who were older and had been in a relationship for longer were more likely to decide based on their surrogate predictions for their partner lends support to Tunney and Ziegler’s model.^3^ Indeed, it predicts that surrogates who are more familiar with the recipient are more likely to take a simulated perspective as they believe it would match the recipient’s preferences. This is an encouraging result as these demographic groups are more likely to find themselves having to make a surrogate decision for their partner. However, we did not find that surrogates’ previous discussions with their partner or knowledge of their partner’s preferences increased the likelihood of a simulated decision. This is consistent with the finding that surrogates having prior discussions with their next-of-kin does not increase surrogate accuracy.^2^ This means that although prior discussions and increased knowledge might be helpful from the point of view of the decision maker, they might not be the best way to improve the accuracy of surrogate decisions.

Participants were more likely to accept a life-saving treatment, at the risk of impaired quality of life, for their partner than for themselves. Interestingly, this was despite the fact that surrogates predicted their partner’s decisions to be similar to their own. On the other hand, we did find that surrogate predictions were predictive of surrogate decisions, even after controlling for participant’s own choices. It seems to be the case that surrogates do engage in a simulated perspective and take into account the recipient’s wishes, which moderates the statement that surrogates do not follow the substituted judgment standard. Furthermore, we found new evidence relating to the self-other difference. Participants who were more confident that they made the right decision for their partner showed smaller self-other differences—they were less likely to accept more treatment for their partner than for themselves. This could mean that surrogates believe the wrong decision would be to accept more treatment for their partner than themselves to keep that person alive, which is coherent with the idea that the right decision is one that conforms to the substituted judgment standard, according to our participants.

Contrary to our expectations, we did not find that any of our measures related to mortality had an effect on participants’ propensity to accept life-saving treatment, neither for themselves nor for their partner. This is consistent with Batteux et al.,^13^ who found that surrogates reported similar wishes and decision processes despite large variabilities in their propensity to accept life-saving treatment. More research is therefore needed to understand this variability. There are also many aspects of the experience and acceptance of mortality that we did not investigate here, such as how participants reflected on these life events. Exploring these individual differences in more detail might help elucidate the relationship between age and the likelihood of conforming to the substituted judgment standard.

Our findings are consistent with previous qualitative reports that show that discussions and knowledge of the patient’s wishes helped them throughout the process.^14–16^ Surrogates do worry about whether they have made the right decision after the fact,^23^ thereby reinforcing the need for encouraging discussions in light of our findings. Discussions beyond the surrogate-recipient dyad could also help alleviate conflicts between family members, particularly when the family’s wishes prevent the surrogate from respecting the patient’s wishes.^23^ However, other measures could also be put in place that might be easier than altering the communication patterns of all potential surrogates. Recommendations have been made about how clinicians can ease the process. Clinicians who are informative, available for communication, and supportive of surrogates’ decisions have been found to alleviate the burden experienced by surrogates, which seems to put them in a better position to make a decision that they think is right.^23^ Care providers could also be a valuable resource before the fact, by encouraging and facilitating discussions between patients at risk of losing their decision-making capacity and their family members.

## Conclusions and Future Directions

The present study shows that previous discussions between surrogates and the recipient should ease the process of making a surrogate decision and give surrogates more confidence that they are making the right decision but do not increase the likelihood of making a simulated decision and thereby conforming to the substituted judgment standard. Nevertheless, interventions that are designed to foster these discussions between family members would still be useful to relieve the burden placed on the decision maker, particularly for those without previous experiences of illness and death and are therefore less likely to have these discussions. It seems to be the case that older surrogates are more inclined to decide based on their partner’s wishes, although we were not able to disentangle whether this was an effect of age or length of relationship. This would be a fruitful avenue for future research given that older adult partners are far from being the only kind of surrogate-recipient relationship. Indeed, surrogate decisions are often made by adult children of the recipient,^16^ meaning that we need to investigate whether our findings are affected by the nature of the surrogate-recipient relationship. For example, partners might prioritize honoring each other’s wishes, whereas adult children might be drawn to the issue of care when deciding for their parents. If this is the case, discussions would be a more effective way to ease the process in the former than the latter. Finally, although we were not able to measure surrogate accuracy, it is a necessary step to examining the applicability of the substituted judgment standard. It would be useful to assess how the likelihood of making a simulated decision affects surrogate accuracy and whether the factors we identified here influence that relationship.

## Supplemental Material

Supplementary_File_1_online_supp – Supplemental material for On the Likelihood of Surrogates Conforming to the Substituted Judgment Standard When Making End-of-Life Decisions for Their Partner Click here for additional data file. Supplemental material, Supplementary_File_1_online_supp for On the Likelihood of Surrogates Conforming to the Substituted Judgment Standard When Making End-of-Life Decisions for Their Partner by Eleonore Batteux, Eamonn Ferguson and Richard J. Tunney in Medical Decision Making

## Supplemental Material

Supplementary_File_2_online_supp – Supplemental material for On the Likelihood of Surrogates Conforming to the Substituted Judgment Standard When Making End-of-Life Decisions for Their Partner Click here for additional data file. Supplemental material, Supplementary_File_2_online_supp for On the Likelihood of Surrogates Conforming to the Substituted Judgment Standard When Making End-of-Life Decisions for Their Partner by Eleonore Batteux, Eamonn Ferguson and Richard J. Tunney in Medical Decision Making

## Footnotes

a We asked participants whether they were confident that they made the right decision, without giving them an indication of what the “right” decision might refer to. The conclusions we draw about making the right decision are solely from the subjective perspective of the participant. We do not put forward a position on what might be the “right” decision here.
