# Supplementary material for: On the Likelihood of Surrogates Conforming to the Substituted Judgment Standard When Making End-of-Life Decisions for Their Partner
Source: Med Decis Making. 2019 Jul 29;39(6):651–60. doi: 10.1177/0272989X19862800 (PMC6794635; doi:10.1177/0272989X19862800)
Supplement: Supplementary_File_1_online_supp – Supplemental material for On the Likelihood of Surrogates Conforming to the Substituted Judgment Standard When Making End-of-Life Decisions for Their Partner [file Supplementary_File_1_online_supp.docx]

Supplementary File 1 – Materials

1. WALT instrument

*Decisions for the self*

*Functional impairment.* Think about your current state of health. Now imagine that you are suddenly sick with an illness that requires you to be in the hospital for weeks to months. In the hospital, you are offered treatment. The treatment includes many minor tests, such as x-rays, blood draws and CT scans. You also need major therapies such as being in the intensive care unit, receiving surgery, or having a breathing machine. Now imagine that at the end of the treatment, you would be in a state where you would be bedbound. You would not be able to get up out of bed to the bathroom by yourself, and you would need help with all of your daily activities. Without the treatment, you will not survive. The doctor tells you there is a X% chance that the treatment will work and get you back to your current state of health and a X% chance that it will not work and you will be bedbound. Do you want the treatment?

*Cognitive impairment.* Think about your current state of health. Now imagine that you are suddenly sick with an illness that requires you to be in the hospital for weeks to months. In the hospital, you are offered treatment. The treatment includes many minor tests, such as x-rays, blood draws and CT scans. You also need major therapies such as being in the intensive care unit, receiving surgery, or having a breathing machine. Now imagine that at the end of the treatment, you would be in a state where your mind would not be working, such that you would not be aware of what was going on around you or be able to recognize your loved ones. Without the treatment, you will not survive. The doctor tells you there is a X% chance that the treatment will work and get you back to your current state of health and a X% chance that it will not work and you will be unaware. Do you want the treatment?

*Decisions for partner*

*Functional impairment.* Think about your partner’s current state of health. Now imagine that they are suddenly sick with an illness that requires them to be in the hospital for weeks to months. In the hospital, they are offered treatment. The treatment includes many minor tests, such as x-rays, blood draws and CT scans. They also need major therapies such as being in the intensive care unit, receiving surgery, or having a breathing machine. Now imagine that at the end of the treatment, they would be in a state where they would be bedbound. They would not be able to get up out of bed to the bathroom by themselves, and they would need help with all of their daily activities. Without the treatment, they will not survive. The doctor tells you there is a X% chance that the treatment will work and get your partner back to their current state of health and a X% chance that it will not work and your partner will be bedbound. Would you choose the treatment for them?

*Cognitive impairment.* Think about your partner’s current state of health. Now imagine that they are suddenly sick with an illness that requires them to be in the hospital for weeks to months. In the hospital, they are offered treatment. The treatment includes many minor tests, such as x-rays, blood draws and CT scans. They also need major therapies such as being in the intensive care unit, receiving surgery, or having a breathing machine. Now imagine that at the end of the treatment, they would be in a state where their mind would not be working, such that they would not be aware of what was going on around them or be able to recognize their loved ones. Without the treatment, they will not survive. The doctor tells you there is a X% chance that the treatment will work and get your partner back to their current state of health and a X% chance that it will not work and your partner will be unaware. Would you choose the treatment for them?

1. Questionnaires

**Decision process (scale from 1-5)**

- How confident are you that you made the right decisions for yourself?
- How confident are you that you made the right decisions for your partner?
- Do you feel like you know your partner’s wishes when it comes to these scenarios?
- To what extent have you previously discussed end-of-life scenarios (similar to the ones you were presented with today) with your partner?

**Fear of death scale (scale from 1-5)**

How disturbed or anxious are you by the following aspects of your own death and dying?

- The shortness of life
- Missing out on so much after you die
- Dying young
- The physical degeneration involved
- The pain involved in dying
- The intellectual degeneration of old age
- The uncertainty as to how bravely you will face the process of dying
- Your lack of control over the process of dying
- Leaving the people close to you behind
- The uncertainty of not knowing what happens after death

How disturbed or anxious are you by the following aspects of your partner’s death and dying?

- Losing someone close to you
- Never being able to communicate with the person again
- Regret over not being nicer to the person when they were alive
- Growing old alone without the person
- Feeling lonely without the person
- Having to be with someone who is dying
- Watching the person suffer from pain
- Seeing the physical degeneration of the person’s body
- Not knowing what to do about your grief at losing the person
- Watching the deterioration of the person’s mental abilities

**Life experience checklist (yes/no answer)**

Now we are going to ask you some questions about events in your life that are frightening, upsetting, or stressful to most people. Please think back over your whole life when you answer these questions. Some of these questions may be about upsetting events you don’t usually talk about. Your answers are important, but you do not have to answer any questions that you do not want to. Thank you.

- Have you ever had a very serious accident or accident-related injury (for example, a bad car wreck or an on-the-job accident)?
- Have you ever had a very serious physical or mental illness (for example, cancer, heart attack, serious operation, felt like killing yourself, hospitalised because of nerve problems)?
- Has a very serious accident or accident-related injury (for example, a bad car wreck or an on-the-job accident) ever happened to someone close to you so that even though you didn’t experience it yourself, you were affected by it?
- Has a serious physical or mental illness (for example, cancer, heart attack, serious operation, felt like killing yourself, hospitalised because of nerve problems) ever happened to someone close to you so that even though you didn’t experience it yourself, you were affected by it?
- Have you ever been responsible for taking care of someone close to you who had a severe physical or mental handicap (for example, cancer, stroke, AIDS, nerve problems, can’t hear, see, walk)?
- Has someone close to you died suddenly or unexpectedly (for example, sudden heart attack, murder or suicide)?
- Has someone close to you died (do NOT include those who died suddenly or unexpectedly)?
