# Supplementary material for: On the Likelihood of Surrogates Conforming to the Substituted Judgment Standard When Making End-of-Life Decisions for Their Partner
Source: Med Decis Making. 2019 Jul 29;39(6):651–60. doi: 10.1177/0272989X19862800 (PMC6794635; doi:10.1177/0272989X19862800)
Supplement: Supplementary_File_2_online_supp – Supplemental material for On the Likelihood of Surrogates Conforming to the Substituted Judgment Standard When Making End-of-Life Decisions for Their Partner [file Supplementary_File_2_online_supp.docx]

Supplementary File 2 – Full results of the mediation analyses

*Hypothesis 2c.* The mediation analysis (model 6) with age as IV and self as DV, with experiences and fear of own death as mediator variables, was not significant. The total effect of age on self was not significant (*B*=0.149, *SE*=0.107, *p*=.164). The direct effect of age on experiences was significant (*B*=0.017, *SE*=0.005, *p*=.002) and accounted for 6.48% of the variance in experiences. The direct effects of age (*B*=-0.029, *SE*=0.033, *p*=.375) and experiences (*B*=-0.686, *SE*=0.485, *p*=.159) on fear of own death were not significant (*F_2,150_*=1.838, *p*=.163). The direct effects of age (*B*=0.141, *SE*=0.112, *p*=.208), experiences (*B*=0.213, *SE*=1.66, *p*=.898) and fear of own death (*B*=-0.113, *SE*=0.278, *p*=.686) on self were not significant (*F_3,149_*=0.708, *p*=.579). The indirect effect of age on self was not significant through experiences (effect=0.004 [-0.054, 0.077]) or fear of own death (effect=0.003 [-0.020, 0.039]) or experiences and fear of own death (effect=0.001 [-0.009, 0.012]).

*Hypothesis 2d.* The mediation analysis (model 6) with age as IV and partner as DV, with experiences and fear of partner’s death as mediator variables, was not significant. The total effect of age on partner was not significant (*B*=0.084, *SE*=0.118, *p*=.477). The direct effect of age on experiences was significant (*B*=0.017, *SE*=0.005, *p*=.002) and accounted for 6.48% of the variance in experiences. The direct effects of age (*B*=-0.046, *SE*=0.024, *p*=.056) and experiences (*B*=-0.202, *SE*=0.356, *p*=.573) on fear of partner’s death were not significant (*F_2,150_*=2.443, *p*=.090). The direct effects of age (*B*=0.040, *SE*=0.124, *p*=.747), experiences (*B*=0.930, *SE*=1.82, *p*=.609) and fear of partner’s death (*B*=-0.572, *SE*=0.415, *p*=.170) on partner were not significant (*F_3,149_*=0.913, *p*=.436). The indirect effect of age on partner was not significant through experiences (effect=0.044 [-0.040, 0.149]) or fear of partner’s death (effect=0.016 [-0.054, 0.094]) or experiences and fear of partner’s death (effect=0.002 [-0.008, 0.013]).

*Hypothesis 3b.* The mediation analysis (model 6) with age as IV and simulation as DV, with discussions, knowledge and confidence as mediator variables, was not significant. The total effect of age on simulation was significant (*B*=0.098, *SE*=0.046, *p*=.035) and accounted for 2.92% of the variance. The direct effect of age on discussions was significant (*B*=0.011, *SE*=0.038, *p*=.006) and accounted for 4.94% of the variance in discussions. The direct effect of age on knowledge was not significant (*B*=-0.002, *SE*=0.003, *p*=.599) but discussions were (*B*=0.453, *SE*=0.070, *p*<.001); together they accounted for 22.2% of the variance in knowledge (*F_2,150_*=21.427, *p*<.001). The direct effects of age (*B*=0.003, *SE*=0.003, *p*=.400) and discussions (*B*=0.085, *SE*=0.074, *p*=.251) on confidence were not significant, but knowledge was (*B*=0.402, *SE*=0.076, *p*<.001); together they accounted for 24.1% of the variance in confidence (*F_3,149_*=15.787, *p*<.001). The direct effect of age on simulation was significant (*B*=0.096, *SE*=0.047, *p*=.042), but the direct effects of discussions (*B*=-0.993, *SE*=1.106, *p*=.370), knowledge (*B*=1.159, *SE*=1.123, *p*=.350) and confidence (*B*=1.826, *SE*=1.225, *p*=.138) were not significant; together they accounted for 6.10% of the variance in simulation (*F_4,148_*=2.403, *p*=.052). The indirect effect of age on simulation through discussions, knowledge and confidence was not significant (effect=0.004 [-0.002, 0.013]) and neither were any of the other indirect effects.

*Hypothesis 4.* The mediation analysis (model 6) with length of relationship as the IV and simulation as the DV, with discussions, knowledge and confidence as mediators was not significant. The total effect of length of relationship on simulation was significant (*B*=0.127, *SE*=0.053, *p*=.017) and accounted for 3.71% of the variance. The direct effect of length of relationship on discussions was significant (*B*=0.011, *SE*=0.004, *p*=.011) and accounted for 4.25% of the variance in discussions. The direct effect of length of relationship on knowledge was not significant (*B*=-0.001, *SE*=0.004, *p*=.722) but discussions were (*B*=0.450, *SE*=0.070, *p*<.001); together they accounted for 22.14% of the variance in knowledge (*F_2,150_*=21.33, *p*<.001). The direct effects of length of relationship (*B*=0.002, *SE*=0.004, *p*=.661) and discussions (*B*=0.092, *SE*=0.073, *p*=.213) on confidence were not significant, but knowledge was (*B*=0.400, *SE*=0.076, *p*<.001); together they accounted for 23.86% of the variance in confidence (*F_3,149_*=15.560, *p*<.001). The direct effect of length of relationship on simulation was significant (*B*=0.127, *SE*=0.054, *p*=.019), but the direct effects of discussions (*B*=-1.017, *SE*=1.097, *p*=.355), knowledge (*B*=1.109, *SE*=1.130, *p*=.369) and confidence (*B*=1.895, *SE*=1.217, *p*=.121) were not significant; together they accounted for 6.96% of the variance in simulation (*F_4,148_*=2.768, *p*=.029). The indirect effect of length of relationship on simulation through discussions, knowledge and confidence was not significant (effect=0.004 [-0.002, 0.015]) and neither were any of the other indirect effects.
